# Emergence of Raman Spectroscopy as a Probing Tool for Theranostics

**DOI:** 10.7150/ntno.81936

**Published:** 2023-03-05

**Authors:** Ruchi Singh, Vikas Yadav, Ashish Kumar Dhillon, Arti Sharma, Tripti Ahuja, Soumik Siddhanta

**Affiliations:** Department of Chemistry, Indian Institute of Technology Delhi, Hauz Khas, New Delhi - 110016, India

**Keywords:** Theranostics, plasmonic probes, Raman and surface-enhanced Raman spectroscopy, photothermal therapy, nanomedicine

## Abstract

Although medical advances have increased our grasp of the amazing morphological, genetic, and phenotypic diversity of diseases, there are still significant technological barriers to understanding their complex and dynamic character. Specifically, the complexities of the biological systems throw a diverse set of challenges in developing efficient theranostic tools and methodologies that can probe and treat pathologies. Among several emerging theranostic techniques such as photodynamic therapy, photothermal therapy, magnetic resonance imaging, and computed tomography, Raman spectroscopy (RS) is emerging as a promising tool that is a label-free, cost-effective, and non-destructive technique. It can also provide real-time diagnostic information and can employ multimodal probes for detection and therapy. These attributes make it a perfect candidate for the analytical counterpart of the existing theranostic probes. The use of biocompatible nanomaterials for the fabrication of Raman probes provides rich structural information about the biological molecules, cells, and tissues and highly sensitive information down to single-molecule levels when integrated with advanced RS tools. This review discusses the fundamentals of Raman spectroscopic tools such as surface-enhanced Raman spectroscopy and Resonance Raman spectroscopy, their variants, and the associated theranostic applications. Besides the advantages, the current limitations, and future challenges of using RS in disease diagnosis and therapy have also been discussed.

## Introduction

Theranostics is a combination of techniques involving diagnostic tests in conjunction with ad hoc medication. It relies on spectroscopic tools for diagnosis and has acquired importance in personalized medicine, particularly in curing fatal diseases 1. Theranostic approaches have been primarily introduced and developed for early-stage detection and prevention of complex diseases that impose a significant economic and emotional burden on the patient population. According to the World Health Organization (WHO), cancer is a leading cause of death and a report by GLOBOCAN2020 indicates that the socioeconomic burden generated by cancer is expected to be shared by 28.4 million patients globally by 2040 2, 3. A diagnostic test combined with an appropriate therapy may allow clinicians to modify the treatment plan based on real-time diagnostic data, making it more suited to the patients being studied 4, 5. Therefore, successful therapeutic and diagnostic management necessitates carefully selecting compatible theranostic methods.

Traditionally, fluorescent probes have been used widely in theranostics to probe living systems with better spatiotemporal resolution 6. However, photobleaching, phototoxicity, and the presence of bulkier fluorescent moieties limit their use in spectroscopy and imaging 6. Similarly, other existing techniques, such as computed tomography (CT), photodynamic therapy (PDT), photothermal therapy (PTT), magnetic resonance imaging (MRI), *etc.,* can be modified and integrated with other suitable probes for theranostic applications. However, the photosensitizers employed in PDT, stay in the blood for a considerable amount of time and might cause phototoxicity when exposed to light. Similarly, the heat used in PDT can damage normal tissues, too, thus limiting their use 7. Whereas, Raman spectroscopy (RS), an optical spectroscopy tool, provides a non-invasive way of studying biochemical and molecular processes without adversely affecting the biological system. RS is currently being explored in theranostics along with its non-linear and plasmon-enhanced counterparts 8, 9. Compared to fluorescence and other bio-imaging methods, RS has a number of advantages, including the ability to use very stable molecular probes, a shorter spectrum bandwidth, a high signal-to-noise ratio, and does not require labels 10. It has been used for the early diagnosis and treatment of various neurodegenerative diseases such as Alzheimer's and Parkinson's 11 12. Besides conventional tissue biopsies or investigations, RS has been utilized for four primary purposes. Firstly, it has a relatively high capability of predicting changes inside complex biomolecules and biological systems effectively. Since cell biochemistry is dynamic and constantly evolving, RS provides a practical approach to identifying the vibrational fingerprints of living cells in real-time. Using correlation spectra and chemometric techniques, it can also distinguish between diseased and normal tissues under *in vivo* and *in vitro* conditions 13 14. Secondly, the scattering cross-section of water is very low and does not interfere with the spectra of bioactive molecules in physiological conditions. The third salient point of RS is the ability to probe a wide range of samples with distinct physical states (bulk, surface, solid, liquid, particles), variable size ranges (from centimeters to nanometres), and various biological samples simultaneously. Finally, it can provide real-time biological information of discriminated cellular components and high-resolution imaging at a lower cost than other biomedical imaging techniques like ultrasound imaging and MRI. Moreover, RS can be coupled with nanoparticle-based therapeutic agents, making it a viable alternative to fluorescence spectroscopy in theranostics.

The efficiency of RS in probing deeper tissues and performing bio-imaging with high sensitivity has enabled it to be used for the early diagnosis of pathological conditions 2. Some variants of RS include Surface-enhanced Raman spectroscopy (SERS), and Resonance Raman spectroscopy (RRS) 15-17. These variants of RS are categorized as linear and non-linear processes. Coherent anti-Stokes Raman spectroscopy (CARS), a non-linear Raman microspectroscopic technique, has a better sensitivity than conventional RS. However, a non-resonant background in the CARS spectrum of biomolecules complicates the analysis and hinders accurate interpretation 15, 16, 18, 19. Other techniques, such as spatially offset Raman spectroscopy (SORS), has the potential to probe living tissues through a depth of 5 cm, facilitating enhanced sensitivity up to 2 orders of magnitude in comparison to conventional RS 20,21. The plasmon-enhanced counterpart, popularly known as SERS, is an effective tool for analyzing the vibrational characteristics of analytes at low concentrations upto single molecule levels 22 23. Moreover, its ability to identify unique fingerprint regions in individual cells and tissues makes it a promising diagnostic tool 24 25. It has been further employed to detect a variety of disease biomarkers including those of the Human Immune Virus (HIV), several types of cancer, and so on 26 27.

Nanoparticles (NPs) are an integral part of theranostic-based drug delivery. The rational design of nano-probes and substrates is one of the critical factors for optimizing theranostic applications 28. Some of the most efficient NP probes are magnetic NPs (MagNPs), plasmon-based gold and silver NPs, silica NPs, and polymeric NPs. Other options include nanocarbon materials and metal oxide NPs, which due to their ease of surface functionalization, have emerged as excellent probes for numerous biological applications 29, such as imaging 30, drug transport 31, and bio-sensing 32. One of the best examples of carbon-based probes is the needle-shaped carbon nanotubes (CNTs) that allow enhanced drug loading with minimal or no effects on the cell penetration process 33-35. Other classes of probes include SERS active nanoparticles and substrates such as metal film on nanoparticle (MFON) that function as an efficient and reproducible plasmonic substrate widely used in bioimaging, chemical sensing, bioanalysis, etc. 36-41. Similarly, a class of NPs known as Janus NPs possesses multiple properties in the same particle. Due to their intrinsic anisotropy, they have attracted great interest in imaging, nanocarrier fabrication, cancer therapy, and theranostics 42. In theranostic applications, where the photothermal application has been the preferred therapy, many morphologies of SERS probes, such as rods, spheres, nanostars, etc., have been employed. Therefore, the combination of NP-based probes destroys not only diseased cells using PTT or facilitates targeted drug release but also provides simultaneous diagnostic multimodal information that can be achieved using Raman-based strategies 43, 44.

In this review, we have discussed the utilization of RS and its variants for theranostic applications. We have highlighted the fabrication strategies and applicability of different Raman probes such as plasmonic NPs, molecular probes, carbon-based nanomaterials, and metal oxide semiconductors for PTT, sensing, and bioimaging.

## Principles of Raman Spectroscopy, its Variants, and their Preliminary Clinical Applications

**Figure 1:**
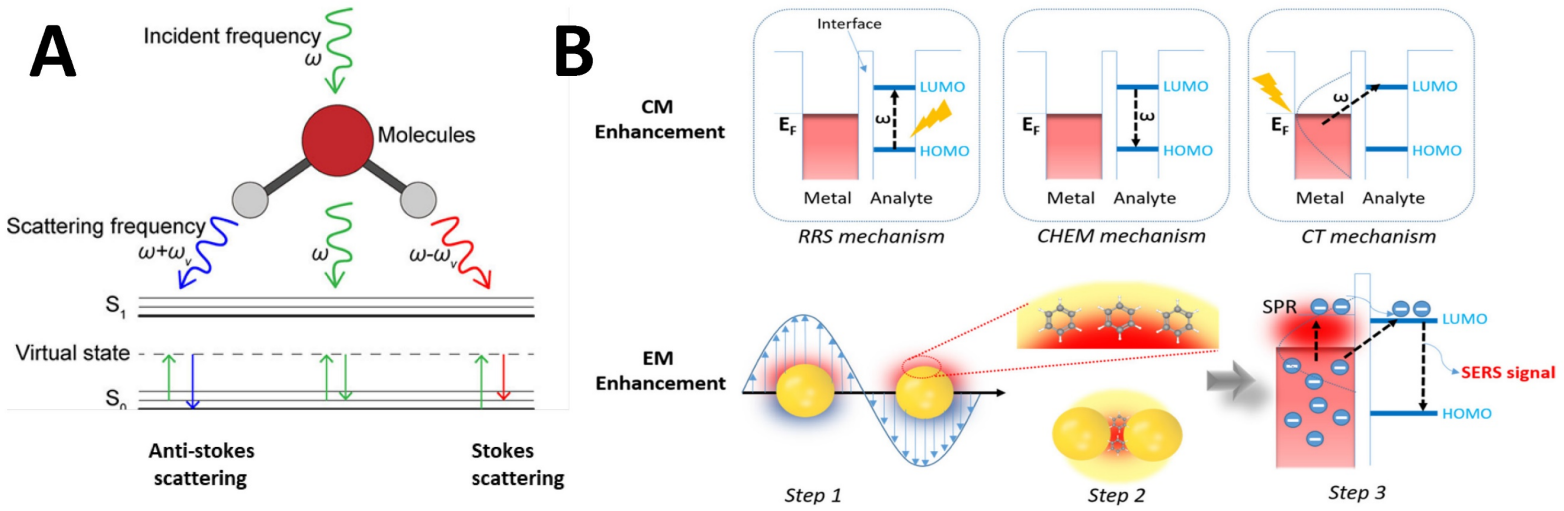
(A) Schematic representation of Raman scattering phenomenon. Figure reprinted from 47 with permission. Copyright (2021) Journal of Applied Physics. (B) Mechanism of enhanced SERS signal of analyte on the noble metal substrate due to electromagnetic and chemical field enhancements. Figure reprinted from 59 with permission. Copyright (2022) The Journal of Physical Chemistry C.

RS is a non-destructive analytical tool that can examine various materials with exquisite molecular specificity 45. It is an inelastic scattering process where electromagnetic radiation strikes the sample, leading to an increase or decrease in the energy of scattered light 46. The inelastic scattering process is called Stokes scattering when the outgoing photon's energy is less than the incident photons, and anti-Stokes when the outgoing photon's energy is more than the incident one (**Figure 1A**) 47. The difference between the incoming and inelastically scattered photon's energy is termed as "Raman shift." The main limitation of RS is that approximately one in 10^6^ photons undergo inelastic scattering, which makes it a weak phenomenon. The following equation gives the Raman scattered intensity (I_R_) in terms of the polarizability factor 48.



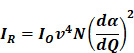



Where I_o_ is the intensity of incident radiation and 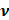 is the frequency of the incoming radiation, N is the number of molecules scattered, and dα/dQ represents the change in polarization of the molecular vibrations. The advent of lasers and the development of optics have greatly improved the performance of Raman spectrometers allowing them to be used in biology and medicine. However, using laser excitation of higher power to achieve a good signal-to-noise ratio can increase the possibility of photodecomposition and degradation of the sample. Additionally, the scattered light can be accompanied by electronic excitations leading to fluorescence emission. This emission mostly interferes with the Raman signals and often appears as background noise in a biological system 49,50. In comparison to other advance spectrometric techniques, Raman techniques such as RRS, Tip-enhanced Raman spectroscopy, and SERS enhance the weak Raman signals and reduce the fluorescence background 51-54. One of the variants of RS suitable for applications in biology and medicine is SERS which was first discovered by Fleischmann *et al.* 55 in 1974 where they observed the enhancement of Raman signals from pyridine molecules electrochemically adsorbed on the surface of the plasmonic silver nanoparticles (AgNPs) 56. Later, Albrecht, Creighton, Jeanmarie, and van Duyne explained the reason for the signal enhancement in SERS, originating from strong electromagnetic fields at the metal surface (Jeanmaire and van Duyne) or by the formation of a molecule-metal complex (Creighton and Albrecht) 57 58. Thus, the SERS enhancement can be broadly classified as an electromagnetic enhancement (EME) or chemical enhancement (CE). **Electromagnetic enhancement (EME):** Such enhancement occurs due to the localization of the electromagnetic field on the surface of the NPs. The enhancement factor contributed by EME is strong and equivalent to 10^10^-10^12^ times the total SERS enhancement. EME is independent of the type of molecule. In this enhancement, direct chemical contact of molecules with the plasmonic substrate is not required; however, the molecules should not be far from the substrate, i.e., the acceptable gap between a molecule and NP is 1-10 nm. Hence such enhancements are considered long-range effects. In EME, the spatial localized regions with high electric fields, termed hotspots, play an important role in the enhancement **Figure 1B** (bottom) 59. In EME, two processes occur simultaneously, i.e., local field enhancement followed by re-radiation enhancement. The EME of a single molecule can be calculated using the below equation:



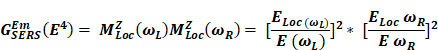



Where, 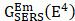 corresponds to electromagnetic enhancement factor, 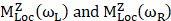 are the field enhancements generated by a laser polarized along Z at the laser and at the Raman frequency, respectively, ω_L_ and ω_R_ attributes to laser and Raman angular frequencies, respectively.

**Chemical Enhancement (CE)**: The formation of NP-molecule complexes results in chemical enhancement **Figure 1B** (top). CE contributes 10^2^-10^3^ orders of magnitude enhancement to total SERS and depends on the type of molecule involved in SERS. In CE, a direct interaction of the molecule with NPs is required; therefore, such enhancement is termed as a short-range effect. The CE can be defined using the below equation:



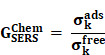



Where 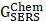 corresponds to the chemical enhancement factor, 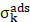 and 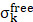 attribute to the Raman cross-section of the adsorbed molecule and free molecule, respectively. Both the enhancement factors combine to give the total SERS enhancement factor. Moreover, the power of total SERS is equivalent to the product of SERS enhancement (EME and CE) and the scattered power of Raman (P_Raman_). A detailed explanation of the mechanisms of SERS enhancement is given in the article of Roberto Pilot et al. 60. Intense Raman signals are observed due to the Localized surface plasmon resonance (LSPR) of the NPs and the resultant lightning rod effect 61-63. For RS variants like SERS, RRS, *etc.*, the incorporation of the metallic NPs makes it possible for their employment in theranostics, where photothermal therapy is used. Both Raman and SERS offer tremendous flexibility in instrumentation and can be portable for field applications 64, 65. Portable SERS devices can enable fast point-of-care (POC) diagnostics 66, 67. Moreover, when used in conjunction with genetic probes, SERS enables multiplex and ultrasensitive detection of nucleic acids produced from tumor, opening up numerous possibilities for early diagnosis and subtyping of clinical malignancy 68, 69.

Another variant of RS is RRS which has not yet been fully explored in the area of theranostics. In this technique, the incident radiation frequency should match with the electronic excitation of molecules, resulting in a 10^6^-fold increase in the Raman signals 70, 71. RRS assists in studying chromophores found in proteins, including chlorophyll carotenoids and iron clusters 72 73. Only a minority of the stretching vibrations of the analyte molecule are amplified under resonance conditions. RRS does not provide information about functional groups that are not involved in the electronic excitation of the molecule, as in the case of molecules having no chromophore groups 74, 75. Hence, RRS is an alternative technique to study the vibrational modes of specific molecules containing chromophores 70 and can be tuned to reveal information about the 'biologically important' fraction of molecules.

In general, RS and its spectroscopic counterparts provide a better demonstration of numerous biomarkers associated with a particular disease, achieving a breakthrough in cell and tissue imaging 76. The efficacy of RS was compared and analyzed by Harada and Takamatsu 77, Kendall *et al.* 78, and Almond *et al.* 79, respectively, for probing of dysplasia in Barrett's oesophagus. They discovered that RS had an 86 percent sensitivity and an 88 percent specificity in distinguishing high-grade dysplasia from adenocarcinoma *in vitro* 79. Lakshmi *et al.* 80 have found a minor loss of lipids in ligamentous sites with no osteoradionecrosis and a complete loss of lipids in osteoradionecrosis bone for oral cancer patients treated with laser radiation therapy. Vidyasagar *et al.* 81 discovered spectral alteration in cervix cancer patients related to radiotherapy responses. Although minimal alterations in Raman spectra were noticed during radiotherapy, it was recognized that higher-resolution spectra could improve the analysis of results. Morris *et al.* 82 used Raman spectroscopic analysis to investigate the long-term effects of radiation on bone chemistry. Radiation-induced breakdown of collagen properties has been found in human cortical bone 83. The Raman spectral features of radiation-induced bone mineral abnormalities were detectable after one week of irradiation that lasted for 26 weeks 80. These unique diagnostic capabilities of RS, if combined with desirable therapeutic probes, can lead to the development of promising theranostic tools for the early diagnosis and treatment of various diseases.

## Raman Probes for Theranostics

**Figure 2:**
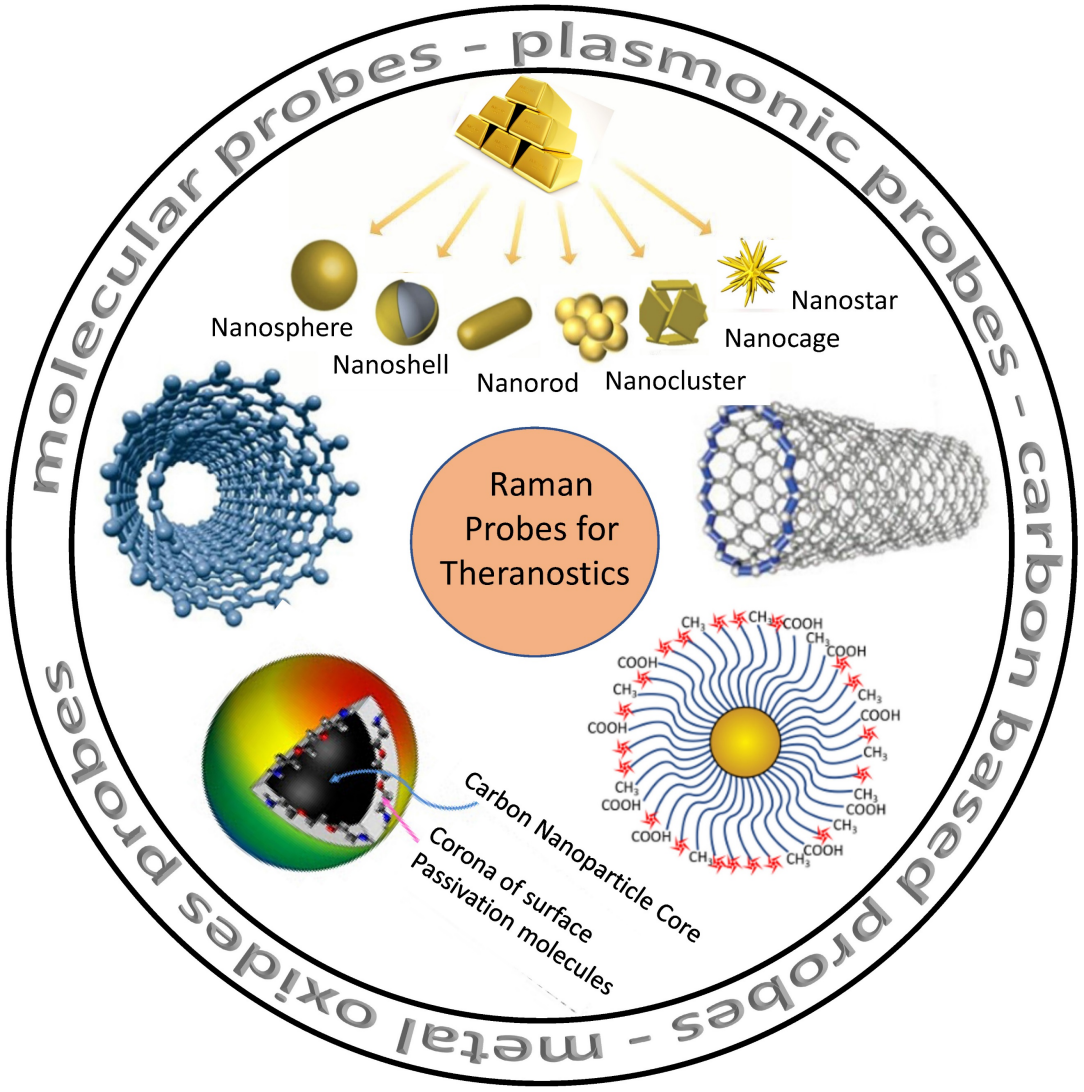
Various probes employed for Raman theranostics such as molecular, plasmonic, carbon, and metal oxide probes. Figure adapted from 84,85,86 with permission. Copyright (2019) Journal of Applied Physics, (2015) Frontiers in Chemistry, (2018) Jove-Journal of Visualized Experiments.

Nanomaterials with RS could provide a brand-new optical detection and imaging technique. As seen in **Figure 2**, such nanomaterials serve as potential Raman probes 84-86. Raman probes are tiny Raman-active moieties with high scattering cross-sections and can target, detect, and image biological entities, such as live cells, tissues, *etc*. 87. Such probes can be of different types: molecular probes, plasmonic probes, carbon-based probes, metal oxides probes, and others, which can be used for concurrent therapy **(Figure 2)**. Using these functionalized probes for targeting specific biomarkers, Zavaleta *et al.* and Palonpon *et al.* successfully demonstrated multimodal imaging in living subjects 88 89. Using a fiber-optic setup, Horgan *et al.* created a theranostic technique that allows *in vitro* real-time cancer diagnosis 90. Such fiber optic setup will make it possible to side-step myriad faults in the current theranostic systems. Shamjith *et al.* 91 reported a quinoline-appended Iridium complex (QAIC) in the form of a molecular probe to be used in fluorescence and SERS modalities for the detection of nicotinamide adenine dinucleotide (NADH) that helps in the regulation of cellular metabolism. The detection limit of NADH was observed at 25.6 nM using luminescence and 15 pM for SERS. When exposed to endogenous NADH, QAIC showed a discernible, time-dependent luminescence turn-ON response 91. Joseph *et al.* have successfully eradicated cancer cells and cancer stem cells using targeted theranostic nanovehicle (TTNV). Raman fingerprint data was specifically used to study biochemical and structural changes in response to various treatments for TTNV 92. The results suggested that the synthesized multipurpose nanocarrier substrate may improve PDT and Photosensitizer Fluorescence Detection (PFD) of gastric cancer tumors *in vitro*. In clinics, nanocarriers show much promise as theranostic agents for treating stomach cancer patients. Thus, Raman probes with tailored properties can lead to the simplification of existing theranostic tools. Some of these advanced Raman probes **(Figure 2)** that have the potential for theranostics are discussed in the sections below.

### Molecular Probes

The development of theranostics depends on molecular probes, which are composed of small molecule moieties and nanosize probes like gold and silver NPs, carbon NPs, MagNPs, semiconducting nanoparticles, small organic molecules, etc. 93, 94. These nanohybrid probes are widely used in theranostics because of easy surface modification, biocompatibility, imaging properties, and exceptional photothermal performance 95. Such molecular probes also serve as efficient Raman reporters/tags. Transition metal-based molecular probes offer unique photophysical properties, long luminescent lifetimes, tunable metal toxicity, and better photostability 96. Due to numerous advantages, these probes are versatile candidates for biomolecular probes 97, 98. Late transition metals are incorporated in the probes, which accounts for the high decay lifespan and reduces the autofluorescence background. Liu *et al.* used bipyridine Ruthenium (II) complex to monitor lysosomal formaldehyde in normal and tumor cells. This Ru (II) complex was reported as an effective tool for the detection of formaldehyde *in vitro* as well as *in vivo* 99. In addition to Ru (II) complex, single molecular probes are used for high-contrast imaging and PTT. Using photoacoustic imaging, Meng *et al*. have created a Hypoxia-triggered single-molecule probe that enables centimeter-level deep tissue penetration and high-contrast visualisation of the tumor 100. In another report, Meng *et al.* have also developed a dual-responsive molecular probe for tumor-targeted imaging and PDT 101. The major benefits of these multifunctional molecular probes is their capacity for simultaneous diagnosis and real-time monitoring of biological processes. Molecular imaging probes can be utilized as non-invasive tools to aid in the early diagnosis of diseases and, more effective treatments 102.

### Carbon-based Probes

**Figure 3:**
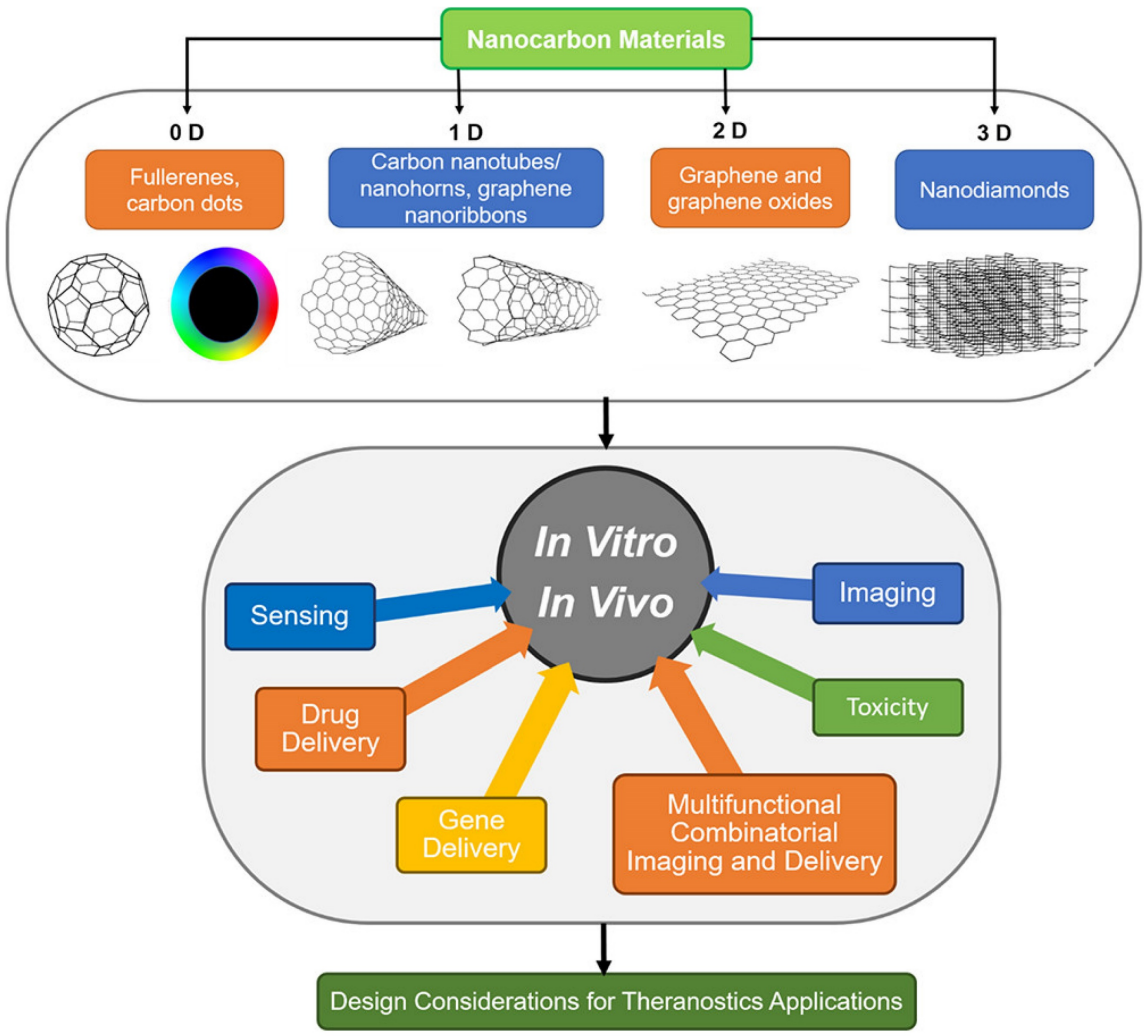
Various types of carbon-based probes with different dimensionality and utilization in theranostics applications. Figure reprinted from 105 with permission. Copyright (2019) Chemical Reviews.

**Figure 4:**
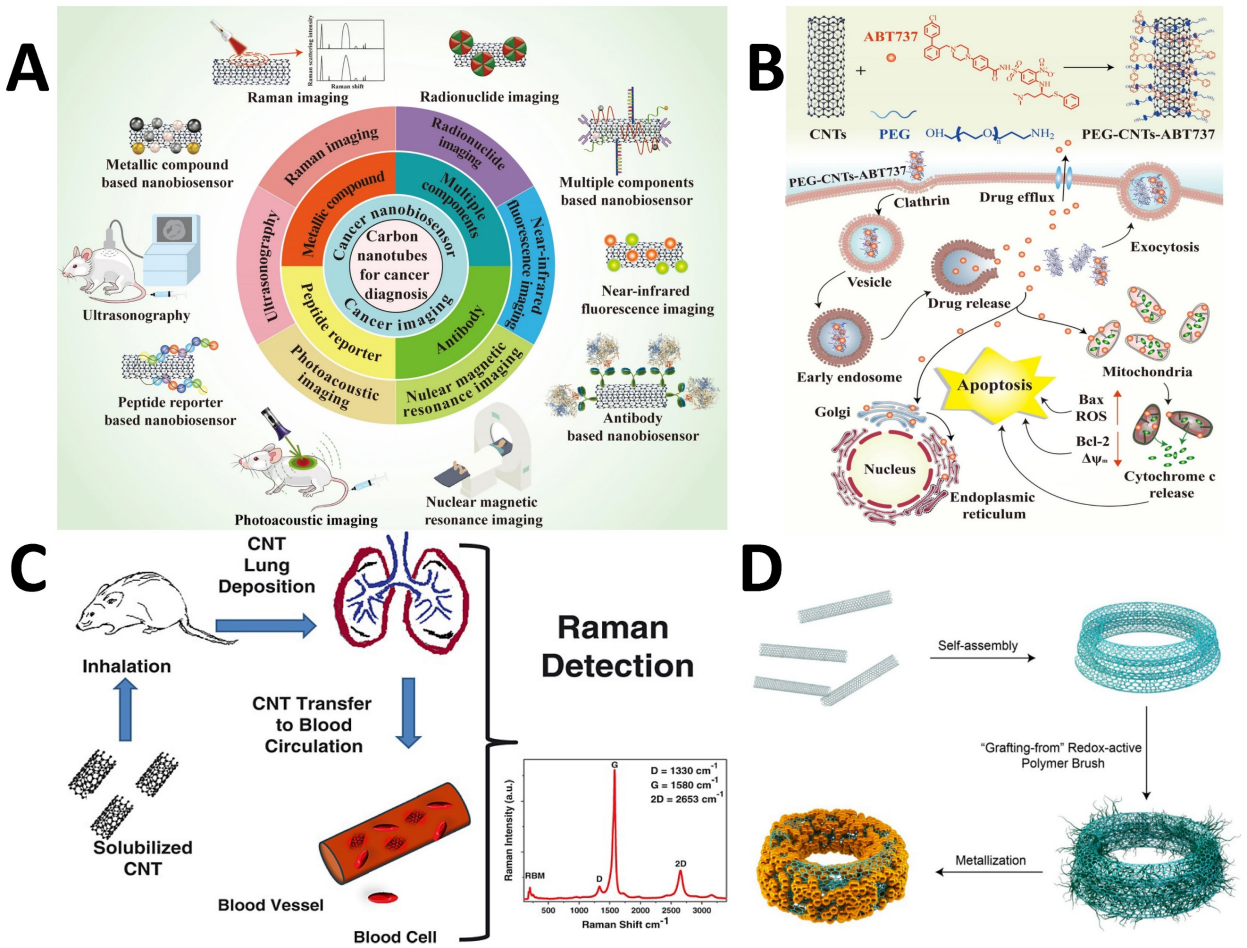
(A) Different applications of CNT for cancer diagnosis. Figure reprinted from 107 with permission. Copyright (2021) Journal of Nanobiotechnology (B) Working mechanism of PEG-CNT-ABT737 for treatment of lung cancer cells. Figure reprinted from 107 with permission. Copyright (2021) Journal of Nanobiotechnology. (C) Mechanism of transfer of CNT to blood circulation for treatment. Figure reprinted from 113 with permission. Copyright (2013) Journal of Applied Toxicology. (D) Self-assembly of CNTs into a CNT ring (CNTR) via redox-active poly(4-vinylphenol) (PvPH) brushes. This utilizes a surface-initiated atom transfer radical-polymerization (SI-ATRP) technique to convert Au^3+^ to Au (0) and encapsulate the CNTR with AuNPs. Figure reprinted from 114 with permission. Copyright (2016) Journal of American Chemical Society.

Because of their excellent mechanical, electrical, thermal, optical, chemical, and peculiar structural dimensional characteristics, carbon-based nanomaterials have attracted a lot of attention in a variety of fields, including biological applications. They are potential candidates for tumor diagnostics due to their distinct Raman spectral features and ease of functionalization. In addition, the intrinsic two-photon fluorescence property in the near-infrared region can facilitate multimodal deep-tissue optical imaging in conjunction with RS 103. According to their size, many carbon-based probe types can be divided into groups. Carbon nanotubes/nanohorns, graphene nanoribbons, fullerene, and carbon nanodots are 0-dimensional (0-D) materials, while graphene and graphene oxides are 2-D and nanodiamonds are 3-D. Several of these nanocarbon probes have been extensively used for biomolecules detection, drug delivery, and imaging of cells and tissues (**Figure 3**) 104 105. The needle-shaped carbon nanotubes (CNTs) with a high aspect ratio allow for more drug loading with minimal or no effects on cell penetration (**Figure 4A**) 33, 106, 107. Robinson *et al.* have investigated the multimodal applications of single-walled carbon nanotubes (SWCNTs) for bioimaging and photothermal treatment of cancer (**Figure 4A**) 107, 108. When coated with PEGylated phospholipids, these probes showed an optical absorbance and characteristic Raman spectra. Kim *et al.* developed a PEGylated CNT-based PEG-CNTs- ABT737 complex. It improved the treatment of lung tissues by specifically targeting mitochondria in malignant cells 109. ABT737 is an inhibitory drug that can decrease the anti-apoptotic protein B-cell lymphoma-2 for the effective treatment of cancer (**Figure 4B**). The application of RS in rapid identification and targeted illumination of CNTs was shown by Bhirde *et al.* 110. In this study, two types of carbon nanotubes (SWCNTs and multi-walled CNTs) were chosen and compared. These carbon nanostructures could transform light into heat because of the electronic transitions in van Hove singularities 111. To promote prolonged dispersion and the best cellular absorption while preventing the harmful effect at lower concentrations, the CNTs were covered with a lipid-polymer coating. RS was used to monitor the CNTs within living ovarian cancerous cells (OVCAR8) using the G and G' bands appearing at 1500-1600 and 2600-2700 cm^-1^, respectively. The significant Raman features of carbon-based probes are D, G, and 2D bands. The D band is due to the disordered structure of graphene corresponding to sp^2^ hybridized carbon atoms. The G band appears at around 1580 cm^-1^ which is due to the E_2g_ mode. It arises due to the stretching of the C-C bond. If there are some randomly distributed impurities or surface charges due to biomolecules, the G peak gets split into two peaks i.e., G peak (1583 cm^-1^) and D' peak (1620 cm^-1^). This is due to the coupling of vibrational modes of impurities with the extended phonon modes of graphene. Nergiz *et al.* investigated the effects of the carbonaceous and metallic nanostructures to create novel multimodal composite nano patches. These composites were made of graphene oxide (GO) layers doped with gold nanostars into the carbon polymeric network for PTT 112. RS has also been employed in conjunction with transmission electron microscopy (TEM) and mass spectrometry to precisely monitor the transport of the graphene-based nano patches within human breast cancer cells, SK-BR-3. Such monitoring was achievable due to distinct Raman signatures of graphene that are defect (D) mode, graphitic (G) band, and the second-order defect mode at 1350, 1560-1590, and 2700 cm^-1^, respectively (**Figure 4C**) 113. The spectral data obtained from the GO-Au nanostar composites revealed peaks with three times increased intensity for the D and G bands compared to pure GO. Song *et al.* designed CNT in an integrated ring (CNTR) configuration through a polymerization redox mechanism and partial doping of AuNP 114. The nanocomposite could be used as a Raman sensor to locate cancerous cells in conjunction with a PA contrast agent to detect a tumor spot (**Figure 4D**) 114. Due to the strong electromagnetic field hotspot, the SERS spectral intensity of CNTR doped with AuNPs was 110 times more enhanced than CNTR. The incorporation of CNTR with the glioblastoma cells in the brain showed that the nanostructure was efficiently absorbed, with overall signal strength increasing with the number of cells. Beqa *et al.* introduced a remarkable nanocomposite by attaching gold nano-popcorn with SWCNTs for targeted diagnosis and PTT of cancer cells 115. PTT was carried out for 10 minutes with a 785 nm laser, which caused a temperature rise of 60°C, killing over 95 percent of the cancer cells. Additionally, intense Raman signals due to D and G bands of SWCNTs hybrids were observed in the tumor-specific areas. This hybrid nanotechnology-based assay was quick since it only took 20 minutes for a cancer cell to connect to the probe and be detected and eliminated. 115. Another type of carbon-based probes, the cucurbiturils (CB[n]s), are gaining popularity due to their unique supramolecular properties, which also make them ideal for precisely managing hot spots of the plasmonic NPs 116. Additionally, Raman-sensitive CB[n]s can selectively trap analytes into the "hotspots" through a variety of interactions, including donor-acceptor, hydrogen bonding, and other interactions that allow for precise molecular recognition. As a result, microfluidics has been used to create SERS substrates that contain CB[n]s functionalized noble metal NPs (NMNPs). Due to the distinct host-guest features of Raman-active substrates, guests might be selectively restricted inside the "hotspot" nanogaps (voids of CB[n]s) for efficient sensing and diagnosis 117. Self-assembled NP probes are also gaining appeal as theranostic agents that are Raman active and paved the way for high-resolution *in vivo* imaging. These probes only form specifically inside cancer cells. Recently, it has been shown that Olsalazine can self-assemble *in vivo* into NPs when conjugated with a benzothiazole compound and a cell-penetrating peptide*.* When it is cleaved by furin, it gets overexpressed in specific cancers 28, 118. In conjunction with NP and carbon-based probes, RS and confocal imaging can identify tumor margins precisely using high-resolution image-based surgery.

### Plasmonic Probes

**Figure 5:**
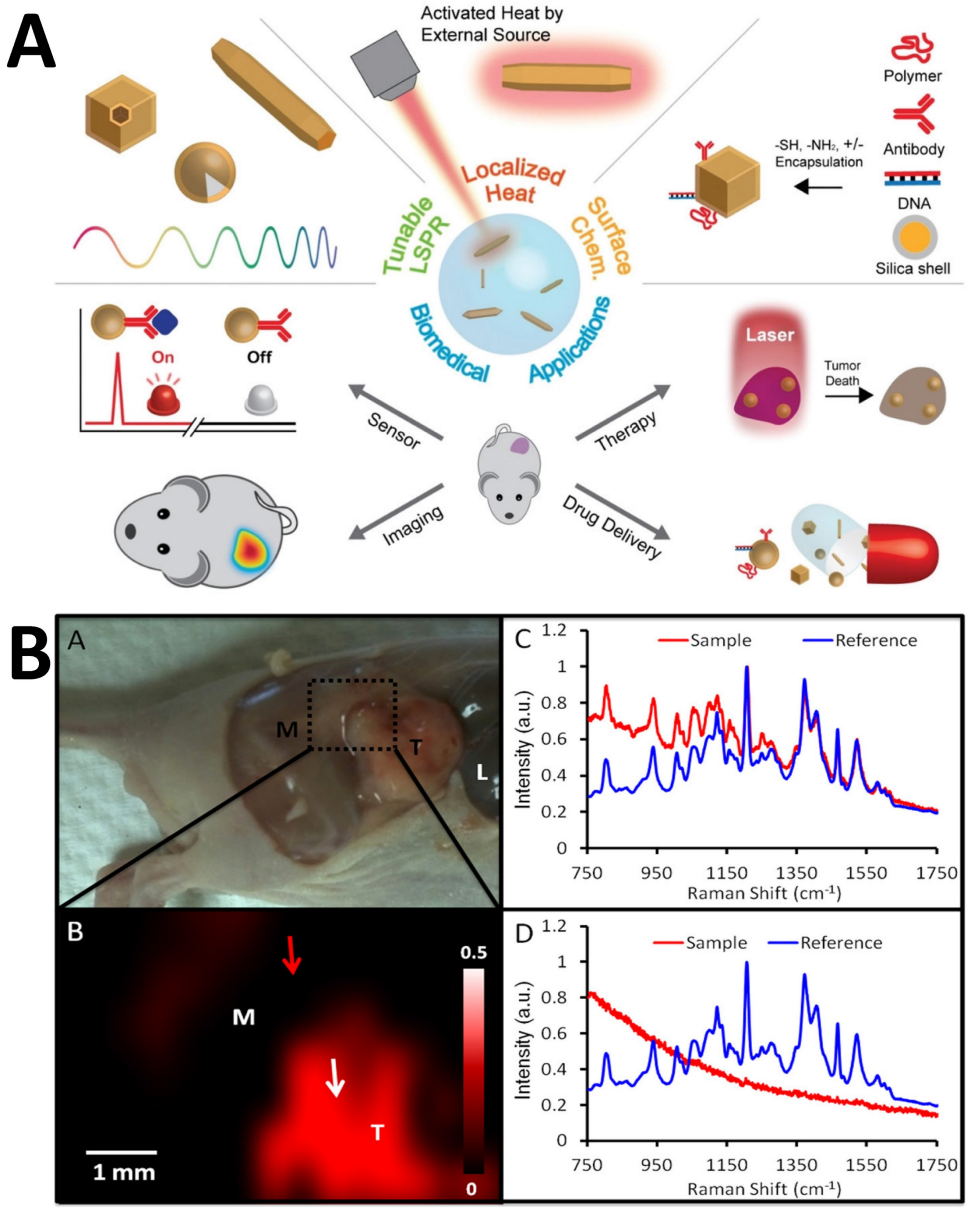
(A) Applications of plasmonic NPs in biomedical applications. Figure reprinted from 123 with permission. Copyright (2019) Advanced Science. (B) SERS imaging for identification of tumor margin. The red part imaging area shows the increment in the tumor. Figure reprinted from 125 with permission. Copyright (2012) ACS Nano.

Noble metals and their nanohybrids, such as core-shell structures, polymeric composites, NPs embedded in 2D materials, etc., are included in the category of plasmonic NPs that are referred to as plasmonic probes. Organic or dye molecules can be attached to the surface of plasmonic nanoparticles that act as SERS tags. Using multiple SERS tags in a probe can lead to efficient multiplexed detection of biological species with low detection limits, high sensitivity, selectivity, and photostability 119, 120. Molecules can be combined with SERS tags to form an immunocomplex whose structure disintegrates and releases Raman probes, causing signal alterations 121. Similarly, SERS-based aptasensors have significantly progressed in the area of food safety 122. To monitor medicine distribution and support PTT and PDT, the NP-Raman combination (manifested through SERS) may be combined with various theranostic techniques (**Figure 5A**) 123.

Zavaleta *et al.* suggested a strategy where they developed Raman endoscopy as an alternative to colonoscopy or conventional endoscopy 124. The researchers proposed a new, flexible, fiber-optic-based Raman spectrometer connected to a single-mode fiber laser that can be added to the endoscopic device to create a multiplexed molecular imaging endoscope. Such a setup does not require any contact and remains free from contamination. A contrast agent made of Au was designed and further divided into batches, each of which was functionalized with ten distinct Raman active compounds. Jokerst *et al.* proposed the potential of coupled PA-SERS to bypass the comparatively modest Raman penetration limit 125. Au nanorods were utilized as the imaging probes that harnessed the enhanced permeability and retention effect for *in situ* identification of ovarian cancers in mice and for determining the subcutaneous margins of tumors (**Figure 5B**) 125. The researchers also proposed that with some specific aspect ratio and Raman active analyte, a massive enhancement in the PA signal was observed, which could persist for more than two days 125. By examining microcalcifications type II with Au@SiO_2_ nanoparticles, Liang and Zhang *et al*. found that SERS, unlike Raman, could easily distinguish benign breast cancer and *ex vivo* premalignant lesions in biopsied breast tissues, normal breast tissues (NB), atypical ductal hyperplasia (ADH), fibroadenoma (FD), invasive ductal carcinoma (IDC), and ductal carcinoma *in situ* (DCIS) 126, 127. Colloidal AgNPs were used by Mert *et al*. to distinguish between malignant and normal cells in biopsied samples taken from the kidneys of healthy individuals as well as those with transitional cell carcinoma (TCC) and renal cell carcinoma, two kinds of kidney cancer (RCC) 128. Feng *et al.* reported that the implantation of AgNPs in nasopharyngeal cancer helps differentiate between two types of carcinomas (C666 and CNE1) from the normal cell lines (NP69) 129. The blood plasma of 34 healthy participants and 32 gastrointestinal cancer patients were detected and spectrally characterized 130. In a separate study, Feng *et al.* demonstrated that the band assignments of SERS spectra revealed some changes in the cancer-specific tissues, comprising exceptionally elevated quantities of nucleic acid, phenylalanine, collagen, phospholipids, a lower proportion of amino acids, and saccharides in the blood plasma of a patient suffering from gastric cancer 131, 132. In comparison to the healthy group, the SERS spectral bands around 725 and 1445 cm^-1^ are elevated for the group with gastric cancer. Ito *et al.* adapted a hexagonal-shaped nanostructure of silver mounted on the surface of a phosphor bronze fabricated chip used during serum investigation of colorectal and gastric cancer 133. They studied the identification of positively-charged blood components such as tumor-associated antigens (TAA), tumor-specific antigens (TSA), and immunoglobulins (Ig) using the negatively charged surface of AgNPs. The blood peak height in SERS spectra was higher in the cancer patient's tissue and continued to rise as the malignancy progressed 133.

**Figure 6:**
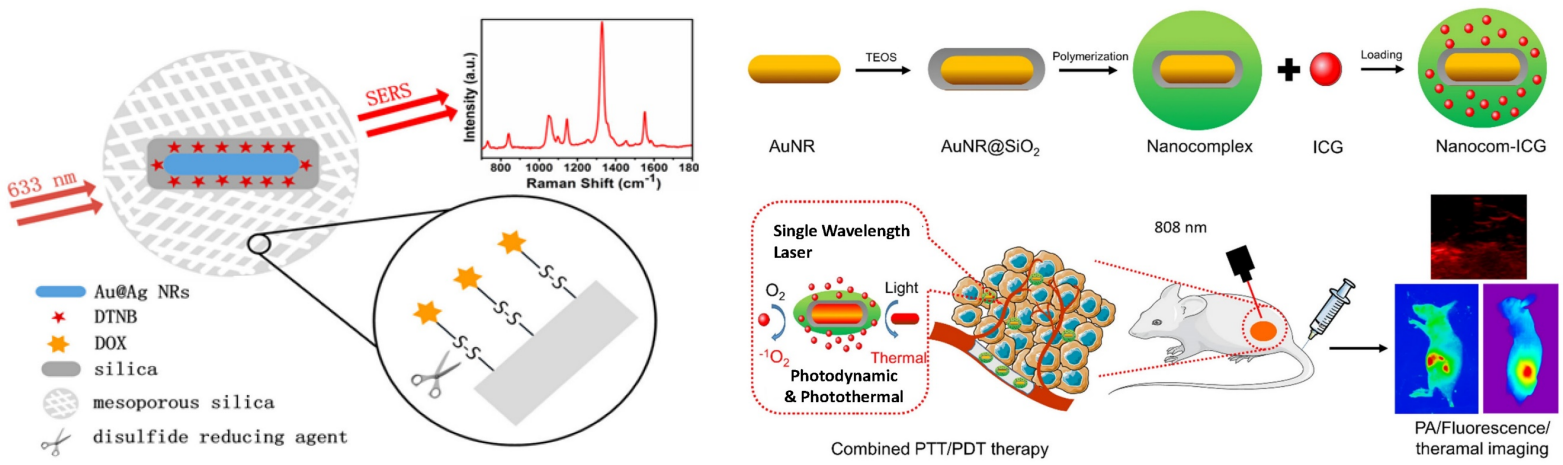
(A) Scheme where disulfide bond acting as a nanocarrier in intracellular drug delivery scheme. Figure reprinted from 138 with permission. Copyright (2013) Analytical Chemistry. (B) Fabrication of Nanocom-ICG in combination for PTT/PDT therapy. Figure reprinted from 142 with permission. Copyright (2021) Journal of Nanobiotechnology.

SERS has been used to screen urine samples for prostate cancer 134. Kneipp *et al.* coupled AuNPs with living cells to get deeper insights into the structure of phenylalanine and DNA in cells 135. Cottat *et al.* utilized Electron-Beam Lithography (EBL) to construct a newly developed SERS technology based on a nano-antenna biosensor made of Au to identify biomarkers in the nano-molar range of concentrations in distinct bio-fluids (serum and saliva) as well as the manganese superoxide dismutase generated by cancer infected liver 136. Madathil *et al.* have fabricated a novel SERS-based catheter device using 30 nm size AgNPs decorated over leaf-like TiO_2_ nanostructures. This device can differentiate between normal, premalignant, and malignant tissues and allows rapid detection, classification, and grading with high accuracy and sensitivity 137. Zong *et al.* employed the SERS technique to understand the mechanism behind the doxorubicin (DOX) released inside the cells by tracking down the pathway of the nanodrug 138. The DOX was covalently linked to the mesoporous silica (MS) through a disulfide linkage, utilizing the principle of tracing Raman/fluorescence signals to identify intracellular drug delivery. The researchers used Au@Ag nanorods labeled with 5,5′ dithiobis-(2-nitrobenzoic acid) (DTNB) and MS to demonstrate the involvement of glutathione (GSH) in DOX release **(Figure 6A)** 138. Lui Z. *et al.* 139 proved the use of Raman for diagnosing tumors through bio-imaging and theranostic applications at the same time without using dye 139. Qian *et al.* 140 reported a scheme in which PEGylated AuNPs were conjugated to tumor-targeting ligand like single-chain variable fragment (ScFv) antibody and systematically injected into mice. Due to their emission in near-infrared windows, they were able to target tumor biomarkers. The difference in SERS intensity by tumor was observed for targeted and non-targeted NPs. This finding demonstrates that EGFR-positive tumors were successfully targeted *in vivo* by the ScFv-conjugated AuNPs 140. Zhang *et al.* constructed a multipurpose plasmonic theranostic substrate for SERS imaging and drug-photothermal treatment using a mesoporous titania-based yolk-shell nanoparticle 141. The idea was to build a single NP that combined the conventional chemotherapy, PTT, and one enhancing reagent for better SERS imaging. Gong *et al.* created a nanoprobe made of thermally sensitive polymer enclosed by AuNRs integrating with incorporating Indocyanine Green (ICG) for tumor treatment 142. The thermal-sensitive polymer coating significantly enhanced drug uptake capacity and also increased ICG stability by boosting ICG J-aggregate generation and promoting targeted ICG deposition in the tumor location. This study suggested that nanocom-improved ICG's therapeutic performance was due to coordinated PTT/PDT therapy following NIR laser irradiation **(Figure 6B)** 142. Several reports by Gambhir and Kircher have demonstrated the use of SERS in multimodal imaging of various pathologies 124, 125, 143, 144. A unique triple-modality MPR nanoparticle has been reported, which stands for magnetic resonance imaging, photoacoustic imaging, and Raman imaging (MPR) that has the potential to delineate the margins of tumor cells up to picomolar sensitivity both *in vivo* as well as *in vitro* using mice as a model. MPR has provided a way to monitor a surgery preoperative as well as in an intraoperative manner. This aids in the tumor diagnosis in a more detailed and extensive manner with better precision 144. SERS has also been used to improve the detection and resection of nonmuscle invasive bladder cancer 145. Interestingly, it was found that passively targeted nanoparticles had a better rate of penetration and concentration in the cancerous human bladder tissues due to enhanced permeability and retention. SERS could clearly delineate between healthy and diseased tissue when antibody-based, and tissue permeability-based targeting mechanisms were used 145. Additionally, transurethral resection (TUR) was guided by multiplexed Raman cystoscopy, and it could detect residual disease, which is generally not observable by traditional white-light cystoscopy 145. A miniature, non-contact opto-electro-mechanical Raman device similar to optical endoscopes has been designed to obtain multiplexed molecular SERS data from patients 143. Photoacoustic imaging can also be used in conjunction with SERS, as demonstrated by Gambhir *et al*. 125, where subcutaneous xenografts of the ovarian cancer cell lines in living mice were imaged with PA imaging and the tumor margin separating cancer and diseased tissue sections could be efficiently delineated by SERS. Core-shell NPs with multimodal capabilities also play a crucial role in diagnostics and therapies. The core-shell NPs of methylene blue-loaded gold nanorod@SiO_2_ (MB-GNR@SiO_2_) were synthesized for medical diagnostics and dual PDT 146. Similarly, Feng *et al.* investigated the bioconjugation of Au nanobipyramids (Au NBPs) with Raman reporter 2-naphthalenethiol (2-NAP) and folic acid to specifically kill and detect MCF-7 breast cancer cells based on photothermal and SERS properties of Au NBPs. This plasmonic substrate allows ultrasensitive *in vitro* detection and *in vivo* SERS imaging of MCF-7 cancer cells 147. Among variable potential plasmonic substrates, gold nanostars (AuNSs), due to their multiple sharp branches, have emerged as 'one for all' plasmonic substrates for cancer theranostics, PA, and photothermal conversion effect 148, 149. Similarly, plasmonic substrate multilayered GNRs are very stable in solutions like NaCl, phosphate-buffered saline (PBS), and culture medium. The stability provides a way to use them for biological purposes, as most biological reactions are in the pH range of 4-8. Contrarily, various imaging methods based on GNRs have been successfully developed, including dark-field scattering imaging, PA, and two-photon luminescence, all of which have quick or even real-time responsiveness in theranostics 150. While the clinically translatable nanoparticle probes typically used in MRI imaging have been studied extensively for their toxicity and biodistribution profiles, such studies are also being conducted for *in vivo* SERS, and their enhanced accumulation in diseased tissues results in obtaining enhanced biochemical signatures for diagnostic and therapeutic purposes 151, 152.

### Metal Oxide Probes

**Figure 7:**
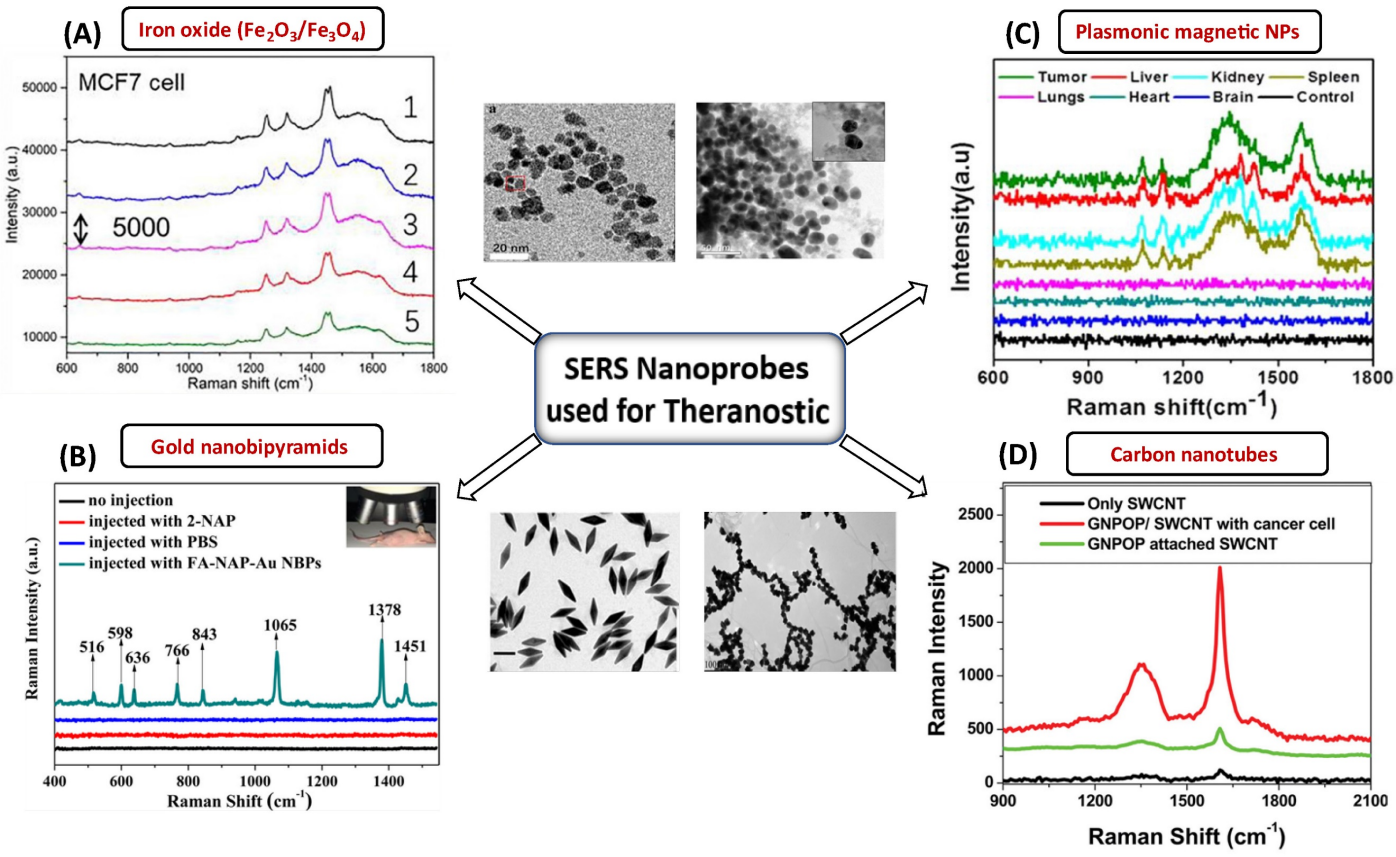
SERS nanomaterials used for the diagnostic purposes (A) Iron-oxide NPs to differentiate between cancerous and normal cells collected from 5 different spots laser in rabbit blood samples. The significant Raman signature bands are at around 1255 cm^-1^ (C=O stretching) and 1325 cm^-1^ (CC stretching) observed for targeted tumor cells. Figure reprinted from 158 with permission. Copyright (2022) Fundamental Research. (B) The detection of MCF-7 cancer cells based on SERS properties of conjugated Au NBPs. The prominent bands are observed due to binding of these AuNBPs with MCF-7 cancer cells. Figure reprinted from 147 with permission. Copyright (2017) ACS Biomaterials Science & Engineering. (C) Plasmonic magnetic NPs for intensity profiling of various organs in tumor areas. The spectral intensities at 1330 and 1090 cm^-1^ are enhanced for tumor targeted areas. Figure reprinted from 166 with permission. Copyright (2021) ACS Applied Bio Materials. D) SERS based cancer cell detection using gold nano-popcorn with SWCNTs. Significant Raman bands at 1300 and 1590 cm^-1^ that correspond to the (D and G) band of SWCNTs were observed for malignant tissues. Figure reprinted from 115 with permission. Copyright (2011) ACS Applied Materials & Interfaces.

Metal oxides are the most intensively explored semiconducting materials, such as Fe_3_O_4_, Fe_2_O_3_, MnFe_2_O_4_, and CoFe_2_O_4,_ for multiplexed applications in MRI, drug delivery*, etc.* 153. In this regard, Yamada *et al.* 154 have investigated the crystals of TiO_2_ (001) and NiO (110) using SERS 155. They described that the charge transfer of the adsorbate-adsorbent interactions resulted in an amplification of Raman scattering due to the chemical interaction in the molecular sites of NiO or TiO_2_ and nitrogen of pyridine. Nb_2_O_5_ NPs were discovered to be a new semiconducting SERS substrate that can replace noble metals and has a number of applications in biology 156. The chemisorption of dye on Nb_2_O_5_ resulted in forming new bonding and antibonding molecular orbitals that can facilitate charge transfer more efficiently with longer excitation wavelengths. Other NPs, such as tungsten oxide, also have interesting properties which can be harnessed for drug delivery, PDT, and PTT 142. *In vitro* and *in vivo* studies have already been conducted using substoichiometric tungsten oxide (WOx) nanostructures coated with biocompatible substances 157. Lin *et al.* 158 have optimized Fe_3_O_4_ nanoparticles that possess excellent SERS activity and can probe up to 5*10^-9^ M concentration of crystal violet. They have optimized the design of the Fe_3_O_4_-based bioprobe in such a way that they can perform dual-mode cancer imaging using SERS and MRI 158. With a Fe_3_O_4_-based bioprobe, subtypes of cancer cells were successfully identified with high sensitivity and specificity *in vitro* via high-resolution SERS imaging. The capability to probe a tumor at an early stage using a unique bioprobe with dual-modal imaging capability is crucial for detecting and treating cancers 158. Metal oxides can be used for oncological imaging *in vitro* because they have the potential to show excellent SERS activity, which can be utilized to differentiate between cancerous and normal cells. They can also be used as a high-potential contrast agent for T1-weighted MRI, enabling active-targeted imaging of tumor tissues *in vivo* 158. Among various metal oxides, the NPs of iron oxide are the first generation of nanomaterials approved by the food and drug administration for their use in bioimaging, i.e., MRI, for the treatment of iron deficiency 159. NIR-responsive multifunctional MagNPs with remote-control photothermal ablation have been designed by Abedin *et al.* 160 for the photothermal treatment of breast cancer. These Au-Fe_2_O_3_ NPs were designed for medical imaging and treatment of breast cancer cells. Au was used as a coating material because it could enhance the photothermal effect by introducing surface plasmons in the NIR region 160. The combined effect of surface plasmon resonance and superparamagnetic properties of Fe_2_O_3_ provides a multimodal platform for nano thermal ablation and MRI. Rissi *et al.* 161 observed an interesting effect of doping zinc oxide NPs with aluminum, gallium, and indium on optoelectrical properties. The doping results in enhanced absorption in the near IR region and emission range in green-yellow, orange-red, and blue-violet, depending upon the doping element. At concentrations below 60 g/mL, ZnO NPs doped with Al^3+^, Ga^3+^, and In^3+^ were harmless to tumor cells. Only high doses (60-100 g/mL) of doped NPs revealed considerable toxicity after 24 hours of incubation. The potential of ZnO NPs for cell death in human tumors and healthy cell lines were also investigated *in vitro* 161. Interestingly, ZnO NPs can stop the early leakage of medications under physiological circumstances 162. It can serve as a therapeutic agent since Zn^2+^ produced by the decomposition of NPs can also overcome the tumor's resistance to 5-fluorouracil (5-FU) and control a number of physiological processes that restrict tumor growth 162. The fluorescence of these nanoprobes can also be enhanced by forming plasmonic conjugates, and concurrently, these plasmonic conjugates can enable SERS detection and imaging in addition to the therapeutic effects of the nanoprobes 162. One such example is a study by Han *et al.* 163, where a multifunctional Fe_3_O_4_/Au cluster/shell nanocomposite for the SERS-assisted theranostic method was fabricated. The use of Au with metal oxide was reported for the first time for prostate-specific antigen detection, MRI, and magnetic hyperthermia. Moreover, these Fe_3_O_4_/Au nanoparticles demonstrated superparamagnetic properties, strong r2 relaxivity, uniform magnetic heating action, and low toxicity, providing a non-contact method with fewer side effects compared to an existing traditional theranostic method like chemotherapy or radiotherapy 163. At the same time, the SERS enhancement could unambiguously detect prostate cancer. While MRI can pinpoint a tumor's site, SERS is a good option for diagnosing prostate cancer and elucidating its tumor margin due to its higher resolution 163. A variety of SERS nanoprobes, including plasmonic NPs, Au NBPs, CNTs, and plasmonic magnetic NPs, together with their corresponding TEM images and SERS spectra for the specific detection of malignant cells and tissues are shown in **Figure 7**. In the current generation of cancer medications, achieving real-time therapy response monitoring is of the utmost importance. Therefore, we can infer that incorporating plasmonic materials with metal oxide NPs provides a way for better theranostic intervention in real-time. Such a combination makes them simultaneously suitable for multiple applications such as MRI, magnetic hyperthermia, and SERS.

### Other Probes

There are various probes from the families of MagNPs, quantum dots (QDs), and activity-based probes that can be utilized as theranostic agents in addition to plasmonic, metal oxide, and carbon ones. The use of MagNPs to carry drugs has recently attracted a lot of attention as a viable tool for cancer treatment 164. The core or the shell of NPs can also be magnetic, rendering unique capabilities to these nanoprobes. MagNPs combined with gold or carbon nanomaterials possess enhanced optical properties that can be used for MRI 165. Jibin *et al.* have fabricated a plasmonic magnetic nanoprobe of iron-oxide hybrid nanoparticles decorated with graphene and gold for SERS imaging, self-guided magnetic resonance, chemodynamic therapy (CDT), and PTT 166. This delivery technique has fewer adverse effects than conventional chemotherapeutic medicines and can administer therapeutic agents to specific locations. The therapeutic agents (e.g., doxorubicin, idarubicin, paclitaxel) can be bioconjugated with a polymer coating of MagNPs, followed by their delivery inside the body 167. Cancer-specific antibodies or folic acid can also be added to these multifunctional MagNPs. Following regulatory approval, clinicians can use newly designed devices that combine multifunctional MagNPs to take better therapeutic action for cancer patients 165. Similarly, zinc QDs have been used in the diagnosis of cancer cells. The *in vivo* studies by Xiao *et al.* 162 of these zinc QDs have shown excellent ability to deliver doxorubicin and 5-FU in the tumor microenvironment. As a result, both conventional and sophisticated Raman probes can be employed as effective theranostic probes for the early diagnosis and treatment of disease.

### Activity-based probes (ABPs)

ABPs are a different class of tiny molecules that frequently bind to the target enzyme's active form via covalent binding. The biological target is inhibited and labeled by these ABPs, which have the ability to target a wide range of proteases 168. Cathepsins and proteases, for instance, are used in theranostic procedures. The increased levels and activity of cathepsins in a variety of human cancers make them useful markers for early detection and the identification of therapeutic targets. Yael *et al.* developed small molecule-based quenched activity-based probes that allow both the detection and simultaneous treatment of cancer cells 169. In conclusion, theranostic probes for SERS and Raman can be widely used to deliver and detect therapeutic substances (including ablation and photothermal therapies). Such a system is ideal for theranostics because the nanoprobes have the properties needed to deliver therapeutic agents, selectively and quickly accumulate in diseased tissue, report the biochemical and morphological characteristics of the region, deliver therapeutic agents, biodegrade, and be safe for healthy tissues. The possible clinical applications include therapy, drug delivery, image-guided tumor margin delineation, labeled or label-free identification of malignant tissues, and finally, non-invasive and label-free assessment of the metastatic potential of the tumors 170.

The overall view of the Raman probes, including their pros and cons in various theranostic tools, has been summarized in **Table 1**.

## Mitigating the challenges of the use of Raman and SERS in theranostics

Through the development of suitable instrumentation, it has been possible to incorporate RS in non-invasive and *in vivo* probing of living systems as well. However, many optics-based methods are limited by low light penetration into tissues compared to other imaging techniques, such as MRI, and RS is no exception 171-173. Thus, this technique is most suitable for lesions or neoplasms on the surface or can be accessed endoscopically or intraoperatively. Therefore, Raman's limitation to only probe surfaces despite the proven safety and efficacy of the tool requires further research and development of probes and instrumentation. Advanced Raman instrumentation, such as confocal Raman microscopy, SORS, etc., has aided in imaging with better penetration depths and improved spatial resolution 173. The integration of RS with novel substrates, such as MPR NPs, NIR-active quantum dots, etc., has also enhanced the penetration depths in deep tissue imaging.

RS has an inherently low scattering cross section, much lower than fluorescence 174, 175. The autofluorescence background often makes it challenging to get a discernible spectrum and complicates the Raman spectral analysis under *in vivo* conditions, specifically under slow imaging 176. However, these problems can be overcome by using a near-IR laser which ensures a much lower fluorescence background, and also by using a fast scanning spectrometer which can either do point, line, or raster scanning using a rotating galvo-mirror 177. The use of near-IR lasers also ensures higher tissue penetration depth and minimum absorbance by the biological medium. The laser can also be guided to the appropriate areas of analysis through special light collection probes in the form of needles or endoscopic setups. The problem of scattering cross-section can be resolved through advanced RS techniques where even single-molecule detection is possible, thus bringing it at par with the sensitivity of fluorescence methods 178. These interventions help overcome the limitations of using RS or SERS for *in vivo* applications 179.

The low photon count resulting from low scattering cross-section in RS results in significant noise in the spectrum, particularly in biological systems where low laser power is required to avoid the burning of the samples 177. The background fluctuations generated by the instability of the laser excitation source, background fluorescence signals, or pixel-to-pixel variations in the detector are significant sources of noise and diminish the reproducibility of signals even at large acquisition times 180. Another form of noise is the dark current, a random stream of electrons thermally activated in the semiconductor-based detector 181. These noises can be reduced by optimizing the Raman system's components, specifically the detector's quantum efficiency, and further sampling the signals by an analog-digital converter 182. Decreasing the detector's temperature also helps reduce the thermal noise to a great extent 183. Several data processing techniques, along with software-based denoising techniques which use advanced chemometric methods and machine learning algorithms, can reduce these noises to a great extent 184. Denoising algorithms and spectral smoothening techniques, such as the Savitzky-Golay method, are now routinely integrated with spectral acquisition software 185-188. The use of a reflective substrate for the samples has also been shown to enhance the signal-to-noise ratio (SNR) 189. Additionally, water immersion objectives are often used to prevent damage to biological samples 177. Techniques such as shifted excitation Raman difference spectroscopy (SERDS) can also be used to remove the fluorescence background in RS and have been demonstrated to be effective in *in vivo* imaging of biological samples 190-193.

The data analysis of the sample involving Raman and SERS is not straightforward, particularly in the label-free modality for biological samples. Biochemical species such as proteins, lipids, nucleic acids, carbohydrates, etc. make up biological systems, and the spectra of a cell, tissue, and bacterium may look remarkably similar to inexperienced eyes 177. This makes the application of RS challenging due to lack of specificity and significant spectral overlap of the various biological constitutents. Several chemometric techniques that heavily rely on supervised or unsupervised machine learning algorithms can effectively observe this minor spectra fluctuation. The use of RS in biology has profited from the recent boom in machine learning techniques 194. The usage of Raman tags using Raman reporter often has some intrinsic disadvantages which affect their biocompatibility, involve high cost, offer limited variety, and are often difficult to tag 149. The stability of the tags in physiological conditions is also an issue and might affect the reliability of the data analysis 119, 120. Therefore, for both Raman and SERS, there is an option to employ the label-free modality, which does not involve the use of any exogenous fluorophores. While the nanoparticle itself may be useful for therapy using photothermal effect etc., the plasmonic coating can enable direct SERS data collection from cells and tissues 195, 196. Additionally, SERS commonly suffers from reproducibility of the signals due to the difficulties in controlling the nanoparticle assemblies and the intense electromagnetic intensities in the junctions, also called the “hotspots” 197. The SERS substrates are extremely sensitive and thus are prone to be contaminated by impurities. Impurities such as amorphous carbon, often a side product of laser-induced burning and degradation, have a huge scattering cross-section and can obscure the actual SERS spectra 177. The leaching of ions from these NPs can cause toxicity in biological systems 198. Nanoparticles such as silver, which has a high SERS enhancement capability, can be toxic to cells due to the generation of reactive oxygen species 199. They also tend to denature proteins and cause pores in the cell membrane. Similarly, a few types of carbon nanomaterials can elicit harmful immune and nonimmune responses in the human body and should be used with caution not to affect healthy cells 200. Moreover, the transport of these nanoprobes to the target areas can be achieved by specific surface coatings or by invoking specialized release mechanisms commonly employed in the case of drug delivery.

## Conclusions and Future Perspectives

The theranostic approach for treating diseases constitutes a system where the therapeutic and diagnostic modalities are integrated into a single system. This approach is challenging but promises to provide patient-tailored therapies and, most importantly, improve clinical outcomes. For successful disease management, choosing the appropriate theranostic tools is imperative. Fluorescence imaging, positron emission tomography, MRI, and CT are a few analytical techniques that have been used in theranostics. But using advanced multimodal spectroscopic tools and optical instruments has given theranostics a new direction. Spectroscopic techniques such as fluorescence and Raman spectroscopies have been combined with PTT to provide sufficient complementarity for seamless use. The emergence of RS as a part of the theranostic tool to determine minor biochemical variations across tissues and cells *in vivo* can diagnose fatal diseases. Such detection is now possible because of spectral fingerprint determination of biomolecules, the advanced variants of RS, and functional probes that have improved the spatial and temporal resolution of samples under investigation. A wide range of Raman probes, including plasmonic probes, metal oxide probes, carbon-based probes, etc., are used in diagnostics, facilitating single-molecule level detection and therapy options, offering rich structural information about cells and tissues without affecting the clinical outcome. Several of such prospective Raman-based probes that have been utilised or could be employed for various theranostic applications have been covered in this review. Numerous theranostic medicines based on nanoparticles have already been used in clinical trials, and many more are still in the pre-clinical stages. The use of RS and its sophisticated variants has the potential to push the limits of nanomedicine even further. They may create intelligent, adaptable theranostic platforms that will facilitate early diagnosis and treatment and help avert the onset of many fatal diseases.

## Figures and Tables

**Table 1:** A brief summary of Raman probes with their pros and cons in theranostics

SI.No.	Nanoprobes	Constituents	Techniques used	Pros and Cons	References
1	Carbon based probes	Carbon nanotubes Graphene oxides Fullerene Nanodiamonds	Raman/SERS PTT	PROS: Ease of functionalization, variable morphological transition, biocompatible, economical, PDT CONS: Non-biodegradable, cytotoxicity	104, 105, 107, 108, 110, 111, 112, 114, 115, 117, 118, 120
2	Plasmonic probes	Silver and gold nanoparticles Gold nanorods Core-shell plasmonic NPs	Raman/SERS PA PDT PTT Multimodal imaging	PROS: Inertness, plasmonic properties, easily tunable, biocompatible, ease of functionalization, antimicrobial effects, high molecular specificity, selectivity and photostability, multimodal imaging CONS: Localized heating may cause damage to tissues, cytotoxicity, costly	[44, 122, 123, 128, 129, 130, 131, 132, 137, 139, 140, 142, 147, 148]
3	Metal oxides probes	Fe_2_O_3_ Fe_3_O_4_ TiO_2_ NiO ZnO Nb_2_O_5_	Raman/SERS PDT PTT MRI	PROS: Antibacterial activity, large durability and stability, inherent imaging characteristics, phytogenic properties CONS: Cytotoxicity, low stability, generation of reactive oxygen species	153, 155, 156, 158, 159, 160, 161, 162
4	Transition metal-based probes	Ruthenium Platinum Iron complexes	Fluorescence Luminescence PDT	PROS: Unique photophysical properties, bioimaging, stability, luminescence CONS: Cytotoxicity, heavy metals accumulation in the body	[99, 100, 101, 102, 161]
5	Other probes	Magnetic NPs Quantum dots	Raman/SERS PTT CDT	PROS: High resolution imaging, good contrast agent in MRI, biocompatibility CONS: Prone to aggregation, high cost, poor sensitivity, toxicity, limited by the external magnetic field	35, 163, 164
